# Trend and Geographic Variation in Incidence and Prevalence of Inflammatory Bowel Disease in Regions Across China: A Nationwide Employee Study Between 2013 and 2016

**DOI:** 10.3389/fmed.2022.900251

**Published:** 2022-07-25

**Authors:** Hong Yang, Runing Zhou, Xiaoyin Bai, Mingyue Guo, Gechong Ruan, Li Wang, Jiaming Qian

**Affiliations:** ^1^Department of Gastroenterology, Peking Union Medical College Hospital, Chinese Academy Medical Sciences and Peking Union Medical College, Beijing, China; ^2^Department of Epidemiology and Biostatistics, Institute of Basic Medical Sciences Chinese Academy of Medical Sciences, School of Basic Medicine Peking Union Medical College, Beijing, China

**Keywords:** inflammatory bowel disease, epidemiology, prevalence, incidence, China

## Abstract

**Background:**

Incidence and prevalence rates and trends of inflammatory bowel disease (IBD) in China remain largely unknown.

**Objective:**

This study aimed to estimate the nationwide prevalence and incidence of IBD and identify its noticeable trends in China between 2013 and 2016.

**Methods:**

We conducted a population-based analysis using data from the National Urban Employee Basic Medical Insurance database. Patients with at least three claims of IBD diagnosis were identified. A Joinpoint regression model was used to analyze the annual percent change (APC) of the age-standardized incidence and prevalence.

**Results:**

The age-standardized prevalence of Crohn's disease (CD) increased from 1.59/100,000 in 2013 to 3.39/100,000 (*p* < 0.05) in 2016, and that of ulcerative colitis (UC) increased from 8.72/100,000 to 17.24/100,000 (*p* < 0.05) during the period, with a UC/CD ratio of 5.09 in 2016. The age-standardized incidence of CD varied between 0.82/100,000 and 0.97/100,000 (*p* = 0.9), whereas that of UC slightly increased from 4.54/100,000 to 4.85/100,000 (*p* = 0.7). The eastern region of China had the highest incidence and prevalence, and the western region had the lowest rates, in both UC and CD, showing an east-to-west gradient.

**Conclusion:**

The incidence and prevalence of IBD in most urban regions in China had an emerging trend over the study period, and an east-to-west gradient was observed, which indicated a greater burden in eastern China. Efforts to improve prevention strategies and promote awareness of IBD are needed, particularly in young men who are at higher risk for CD.

## Introduction

Although the incidence of inflammatory bowel disease (IBD) has been stable or decreasing in Western countries after the 1990s, its incidence appears to be increasing in newly industrialized countries in Africa, South America, and Asia, including China (1, 2). The shift may be related to the process of industrialization and the change in environmental factors (3). Sporadic studies have shown that the prevalence and incidence of IBD in China are relatively lower than that in the Western countries (4, 5). However, cases of ulcerative colitis (UC) appear to be increasing gradually based on the hospital-based studies and register study (6, 7). A study in Hong Kong revealed an increasing trend of IBD incidence over the past decades, with the age-standardized incidence increasing from 0.10/100,000 in 1985 to 3.12/100,000 in 2014; the prevalence of Crohn's disease (CD) in 2014 was 24.5/100,000 (8). With the rapid economic development and aging of population in China in recent decades, the temporal and spatial trends in the prevalence and incidence of IBD have attracted much attention. However, these studies are limited in their design and nationwide representation across China, a large country with wide geographic variation in climate, healthcare, industrialization, and urbanization. A nationwide epidemiological study is, therefore, needed to provide a comprehensive assessment of IBD occurrence and trends in China, which will provide a deep understanding of the worldwide epidemic trend of IBD.

The National Urban Employee Basic Medical Insurance (NUEBMI) system was established in 1998. The NUEBMI database collects disease diagnosis, insurance, and claim information of outpatient, emergency, and inpatient care from 30 provincial administrative regions of China. In this study, using this nationwide database, we performed a temporal trend study to estimate sex-, age-, and geographic-specific incidence and prevalence rates of IBD and explore the temporal trend of IBD incidence and prevalence in China.

## Materials and Methods

### Data Source and Study Population

China achieved universal health insurance coverage in 2011, and it has three main insurance systems. One of the three systems, the National Urban Employee Basic Medical Insurance (NUEBMI), was established in 1998 and covers urban employees, including retired employees. The data on IBD patients from 1 January 2013 to 31 December 2016 were obtained from NUEBMI, which is administrated by the China Ministry of Human Resources and Social Security and contains the records of 274.43 to 295.31 million beneficiaries from 30 provincial administrative regions (except Tibet, Hong Kong, Macao, and Taiwan). For this analysis, 7 of the 30 administrative regions (i.e., Beijing, Hebei, Shanghai, Fujian, Tianjin, Gansu, and Ningxia) were excluded due to lack of diagnostic information or reporting issue, which possibly added up false positives. Finally, the data of 150,617,725 beneficiaries overall were extracted from the 23 provincial administrative regions in 2016, accounting for about 10% of the total population of Mainland China (1.38 billion) (9). The database includes the systematic information of outpatients and inpatients, as well as patients in emergency departments, including information about the diagnosis, insurance, and reimbursement.

### Data Extraction of Targeted Patients With UC or CD

To identify patients who had received a diagnosis of UC or CD for the study period, the NUEBMI database was searched with the following key terms in the systematic fields: “main diagnostic name”; “main diagnostic code”; “secondary diagnostic name 1”; “secondary diagnostic code 1”; “secondary diagnostic name 2”; and “secondary diagnostic code 2.” Candidate patients with UC or CD had been claimed at least three times with the diagnostic code K51or K50, as defined by the tenth version of the International Classification of Diseases (ICD-10), or patients were described as UC or CD in the Chinese version of the ICD-10 by considering IBD as chronic relapsing diseases and referencing similar studies using national medical insurance system in order to balance true and false positives. Six specialists in inflammatory bowel diseases (IBD) were independently determined to exclude an uncertain diagnosis. Then, the national ID number of citizens in the reimbursement database was linked to the insurance database, both of which were from NUEBMI, to acquire targeted patients. Meanwhile, the demographic information of patients with UC from the cleaned dataset was collected, including the patient's gender, age, administrative area code, and the date when they visited the physicians. The requirement of informed consent was waived since de-identified aggregated data were used.

### Statistical Analysis

#### Prevalence Rate Estimation

A prevalence case was defined as any (new or existing) patient diagnosed with UC or CD from the NUEBMI database in the corresponding year. Because the NUEBMI database was updated monthly, the insured population of a given year was calculated by dividing the cumulative number of insured persons per month by 12. Prevalence was expressed as cases per 100,000 person-years and was calculated by dividing the number of prevalence cases by the number of insured person-years during a specific year. Age-, sex-, and geographic-specific prevalence rates were calculated. Age-standardized prevalence was calculated using the 2015 Chinese National Population Statistics, which was obtained through the China Information System for Disease Control and Prevention (https://www.chinacdc.cn/). The age and gender composition of NUEBMI individuals and the 2015 Census was rather similar (Supplementary Figure 1).

#### Incidence Rate Estimation

An incidence case was defined as a patient newly diagnosed with UC or CD in the corresponding year. To accurately determine an incidence case, we used a washout period of 1 year since 2012 to exclude patients with a history of healthcare utilization for UC or CD within the year before their claim in the NUEBMI system. The course of the washout period was decided according to other similar studies conducted in Asia [2 years in South Korean studies (10, 11); 6 months in Hong Kong China (12); not mentioned in Taiwan China (13)] and our own investigation on the diagnostic interval of IBD patients, which were around 1 year or less in both CD and UC (unpublished data) and at least 6 months follow-up when the diagnosis was suspected according to Chinese National IBD Guideline.

Incidence was expressed as cases per 100,000 persons and was calculated by dividing the number of incidence cases by the number of insured person-years during a specific year. Age-, sex-, and geographic-specific incidence rates were calculated. Age-standardized incidence was also calculated using the 2015 Chinese National Population Statistics.

#### Statistical Methods

The prevalence and incidence rates were reported with 95% confidence intervals (CIs), assuming a Poisson distribution. The demographic variables included sex, age, and geographic region. All the administrative regions in the analysis were further categorized into three regions (i.e., eastern, central, and western China) according to the recommendation released by the National Bureau of Statistics of China. To examine changes in trends of age-standardized incidence and prevalence, a Joinpoint regression model was used to calculate the annual percent change (APC) between 2013 and 2016.

All statistical analyses were performed using SAS version 9.4 (SAS Institute, Cary, NC, USA) and Joinpoint version 4.7 (National Cancer Institute, Calverton, MD, USA).

#### Ethics Statement

The data underlying this article were provided by the China's Ministry of Human Resources and Social Security with permission. Data will be shared on request to the corresponding author with the permission of China's Ministry of Human Resources and Social Security.

## Results

### Crude and Age-Standardized Prevalence of IBD by Year and Sex

Overall, 24,989 patients with UC were reported in 2016, which is nearly a three-fold increase compared with the 8,683 patients with UC recorded in 2013 (Table 1). The crude prevalence rate of UC, per 100,000, increased from 8.10 (95% CI: 8.00–8.30) in 2013 to 16.60 (95% CI: 16.40–16.80) in 2016. The increasing prevalence of UC occurred in both men and women.

**Table 1:** Annual crude prevalence rate of ulcerative colitis among urban employees in 23 provinces (municipalities, autonomous regions) of China from 2013 to 2016 (per 100,000 persons).

	**Total**		**Male**		**Female**	
	**Num. of patients**	Rate (95%CI^*^)	**Num. of patients**	**Rate (95%CI)**	**Num. of patients**	**Rate (95%CI)**
2013	8,683	8.10 (8.00–8.30)	4,679	7.90 (7.70–8.20)	4,004	8.40 (8.10–8.70)
2014	14,173	11.20 (11.00–11.40)	7,791	11.10 (10.90–11.40)	6,382	11.30 (11.00–11.60)
2015	18,956	14.20 (14.00–14.40)	10,497	14.20 (14.00–14.50)	8,459	14.20 (13.90–14.50)
2016	24,989	16.60 (16.40–16.80)	13,894	16.70 (16.40–17.00)	11,095	16.40 (16.10–16.80)

* *Confidence interval*.

Estimated based on the 2015 Census of China's resident population as the reference, the age-standardized prevalence of UC per 100,000 increased from 8.72 (95% CI: 8.53–8.91) in 2013 to 17.24 (95% CI 17.02–17.46) in 2016, with an annual growth rate of 24.20% (95% CI: 11.90–37.90%, *p* < 0.001). The age-standardized prevalence showed similar trends among both men and women but increased faster among men than women, with average growth rates of 25.80 and 22.00%, respectively (Table 2, Supplementary Figure 2A).

**Table 2:** Annual age-standardized prevalence rate of ulcerative colitis among urban employees in 23 provinces (municipalities, autonomous regions) of China from 2013 to 2016 (per 100,000 persons)^*^.

	**Total**	**Male**	**Female**
2013	8.72 (8.53–8.91)	8.25 (8.01–8.50)	9.41 (9.10–9.72)
2014	11.76 (11.56–11.96)	11.47 (11.21–11.73)	12.22 (11.90–12.53)
2015	14.84 (14.62–15.06)	14.61 (14.32–14.90)	15.19 (14.86–15.53)
2016	17.24 (17.02–17.46)	17.1 (16.81–17.40)	17.47 (17.13–17.81)
APC^**^(%)	24.20 (11.90–37.90)	25.80 (11.70–41.70)	22.00 (11.80–33.00)

* *The age-standardized prevalence rate was estimated based on China's resident population in 2015*.

** *Annual percentage change*.

The crude prevalence rates and total numbers of CD patients from 2013 to 2016 are shown in Table 3. The crude prevalence increased from 1.70/100,000 (95% CI: 1.60–1.70/100,000) in 2013 to 3.50/100,000 (95% CI: 3.40–3.50/100,000) in 2016. Although the prevalence increased in both sexes, the overall rates were significantly higher in men than in women (*p* < 0.001).

**Table 3:** Annual crude prevalence rate of Crohn's disease among urban employees in 23 provinces (municipalities, autonomous regions) of China from 2013 to 2016 (per 100,000 persons).

	**Total**		**Male**		**Female**	
	**Num. of patients**	Rate (95%CI^*^)	**Num. of patients**	**Rate (95%CI)**	**Num. of patients**	**Rate (95%CI)**
2013	1,778	1.70 (1.60–1.70)	1,138	1.90 (1.80–2.00)	640	1.30 (1.20–1.40)
2014	3,004	2.40 (2.30–2.50)	1,917	2.70 (2.60–2.90)	1,087	1.90 (1.80–2.00)
2015	4,016	3.00 (2.90–3.10)	2,558	3.50 (3.30–3.60)	1,458	2.40 (2.30–2.60)
2016	5,281	3.50 (3.40–3.50)	3,390	4.10 (3.90–4.20)	1,891	2.80 (2.70–2.90)

* *Confidence interval*.

We calculated that the overall age-standardized prevalence rates in CD patients increased from 1.59/100,000 (95% CI: 1.51–1.67/100,000) in 2013 to 3.39/100,000 (95% CI: 3.29–3.48/100,000) in 2016, with an APC of 26.30% per year (95% CI: 9.90–46.50%, *p* < 0.05) over the 4-year study period (Table 4, Supplementary Figure 2B). The age-standardized prevalence for men more than doubled, increasing from 1.81/100,000 (95% CI: 1.71–1.93/100,000) in 2013 to 3.92/100,000 (95% CI: 3.78–4.06/100,000) in 2016, with an APC of 26.80% (95% CI: 10.30–46.00%, *p* < 0.05). To a lesser extent, the age-standardized prevalence for women increased from 1.35/100,000 (95% CI: 1.24–1.46/100,000) in 2013 to 2.78/100,000 (95% CI: 2.65–2.91/100,000) in 2017, with an APC of 25.0% (95% CI: 6.20–47.00%, *p* < 0.05).

**Table 4:** Annual age-standardized prevalence rate of Crohn's disease among urban employees in 23 provinces (municipalities, autonomous regions) of China from 2013 to 2016 (per 100,000 persons)^*^.

	**Total**	**Male**	**Female**
2013	1.59 (1.51–1.67)^**^	1.81 (1.71–1.93)	1.35 (1.24–1.46)
2014	2.31 (2.22–2.39)	2.66 (2.52–2.77)	1.93 (1.81–2.05)
2015	2.91 (2.81–3.00)	3.32 (3.18–3.45)	2.45 (2.32–2.59)
2016	3.39 (3.29–3.48)	3.92 (3.78–4.06)	2.78 (2.65–2.91)
APC** (%)	26.30 (8.90–46.50)	26.80 (10.30–46.00)	25.00 (6.20–47.00)

* *The age-standardized prevalence rate was estimated based on China's resident population in 2015*.

** *Annual percentage change*.

The UC/CD ratio in overall age-standardized prevalence was decreasing to 5.48, 5.09, 5.10, and 5.09 each year from 2013 to 2016.

### Crude and Age-Standardized Incidence of IBD by Year and Sex

There were 4,551 new cases of UC in 2013 and 7,147 new cases of UC in 2016. The crude incidence rate of UC fluctuated slightly from 2013 to 2016, in the range of 4.30 to 4.70/100,000 (Table 5). Men had a significantly higher incidence of UC than women, with the annual incidence, per 100,000, being 4.90 (95% CI 4.8–5.1) among men and 4.60 (95% CI 4.40–4.70) among women (*P* = 0.002).

**Table 5:** Annual crude incidence rate of ulcerative colitis among urban employees in 23 provinces (municipalities, autonomous regions) of China from 2013 to 2016 (per 100,000 persons).

	**Total**	**Male**	**Female**			
	**Num. of patients**	Rate (95%CI^*^)	**Num. of patients**	**Rate (95%CI)**	**Num. of patients**	**Rate (95%CI)**
2013	4,551	4.30 (4.10–4.40)	2,528	4.30 (4.10–4.50)	2,023	4.20 (4.10–4.40)
2014	5,740	4.50 (4.40–4.70)	3,276	4.70 (4.50–4.80)	2,464	4.40 (4.20–4.50)
2015	5,740	4.30 (4.20–4.40)	3,233	4.40 (4.20–4.50)	2,507	4.20 (4.00–4.40)
2016	7,147	4.70 (4.60–4.90)	4,076	4.90 (4.80–5.10)	3,071	4.60 (4.40–4.70)

* *Confidence interval*.

The age-standardized incidence appeared to undergo a volatile change from 2013 to 2016 (Table 6, Supplementary Figure 2C). The average annual change was 1.60% (95% CI: −6.70 to 10.70%). The age-standardized incidence of UC per 100,000 for the years 2013, 2014, 2015, and 2016 was 4.54, 4.68, 4.42, and 4.85 (95% CI: 4.40–4.68, 4.56–4.81, 4.30–4.54, and 4.73–4.97), respectively.

**Table 6:** Annual age-standardized incidence rate of ulcerative colitis among urban employees in 23 provinces (municipalities, autonomous regions) of China from 2013 to 2016 (per 100,000 persons)^*^.

	**Total**	**Male**	**Female**
2013	4.54 (4.40–4.68)	4.44 (4.27–4.62)	4.69 (4.47–4.90)
2014	4.68 (4.56–4.81)	4.78 (4.61–4.95)	4.57 (4.39–4.76)
2015	4.42 (4.30–4.54)	4.47 (4.31–4.63)	4.36 (4.19–4.54)
2016	4.85 (4.73–4.97)	4.98 (4.82–5.14)	4.70 (4.53–4.87)
APC^**^ (%)	1.60 (−6.70–10.70)	3.00 (−6.70–13.70)	−0.10 (−8.20–8.70)

* *The age-standardized prevalence rate was estimated based on China's resident population in 2015*.

** *Annual percentage change*.

A total of 4,817 incidence cases of CD were identified in this study. The crude incidence rates and the total number of newly diagnosed CD patients from 2013 to 2016 are shown in Table 7. The overall crude incidence varied in the range of 0.86/100,000 to 0.99/100,000 from 2013 to 2016. The crude incidence rate of CD was significantly higher in men compared with women (*p* < 0.001).

**Table 7:** Annual crude incidence rate of Crohn's disease among urban employees in 23 provinces (municipalities, autonomous regions) of China from 2013 to 2016 (per 100,000 persons).

	**Total**		**Male**		**Female**	
	**Num. of patients**	Rate (95%CI^*^)	**Num. of patients**	**Rate (95%CI)**	**Num. of patients**	**Rate (95%CI)**
2013	919	0.86 (0.81–0.92)	597	1.00 (0.93–1.10)	322	0.67 (0.60–0.75)
2014	1,246	0.99 (0.93–1.00)	790	1.10 (1.10–1.20)	456	0.81 (0.74–0.89)
2015	1,191	0.89 (0.84–0.94)	768	1.00 (0.97–1.10)	423	0.71 (0.64–0.78)
2016	1,461	0.97 (0.92–1.00)	974	1.20 (1.10–1.20)	487	0.72 (0.66–0.79)

* *Confidence interval*.

Age-standardized incidence rates are shown in Table 8 and Supplementary Figure 2D. The overall incidence rates varied between 0.82/100,000 and 0.97/100,000 during 2013–2016, showing no obvious temporal trend (APC = 3.1%, 95% CI: −13.10 to 22.2%, *P* = 0.9). During 2013 through 2016, CD incidence decreased by an average of −1.80% in men (95% CI: −15.70 to 14.3%, *P* = 0.8) and decreased by an average of −0.5% in women (95% CI: −18.10 to 20.90%, *P* = 0.4), but neither change was statistically significant.

**Table 8:** Annual age-standardized incidence rate of Crohn's disease among urban employees in 23 provinces (municipalities, autonomous regions) of China from 2013 to 2016 (per 100,000 persons)^*^.

	**Total**	**Male**	**Female**
2013	0.82 (0.76–0.87)**	0.95 (0.87–1.03)	0.67 (0.59–0.75)
2014	0.97 (0.91–1.02)	1.11 (1.03–1.19)	0.81 (0.73–0.89)
2015	0.85 (0.80–0.90)	0.99 (0.92–1.06)	0.70 (0.63–0.77)
2016	0.95 (0.90–1.00)	0.96 (0.90–1.01)	0.71 (0.64–0.78)
APC^**^ (%)	3.1 (−13.10–22.20)	−1.80 (−15.70–14.30)	−0.50 (−18.10–20.90)

* *The age-standardized prevalence rate was estimated based on China's resident population in 2015*.

** *Annual percentage change*.

The UC/CD ratio in overall age-standardized incidence appeared to decrease from 5.54 to 5.11 in 4 years.

### Age Pattern for IBD Prevalence and Incidence

**Figure 1:**
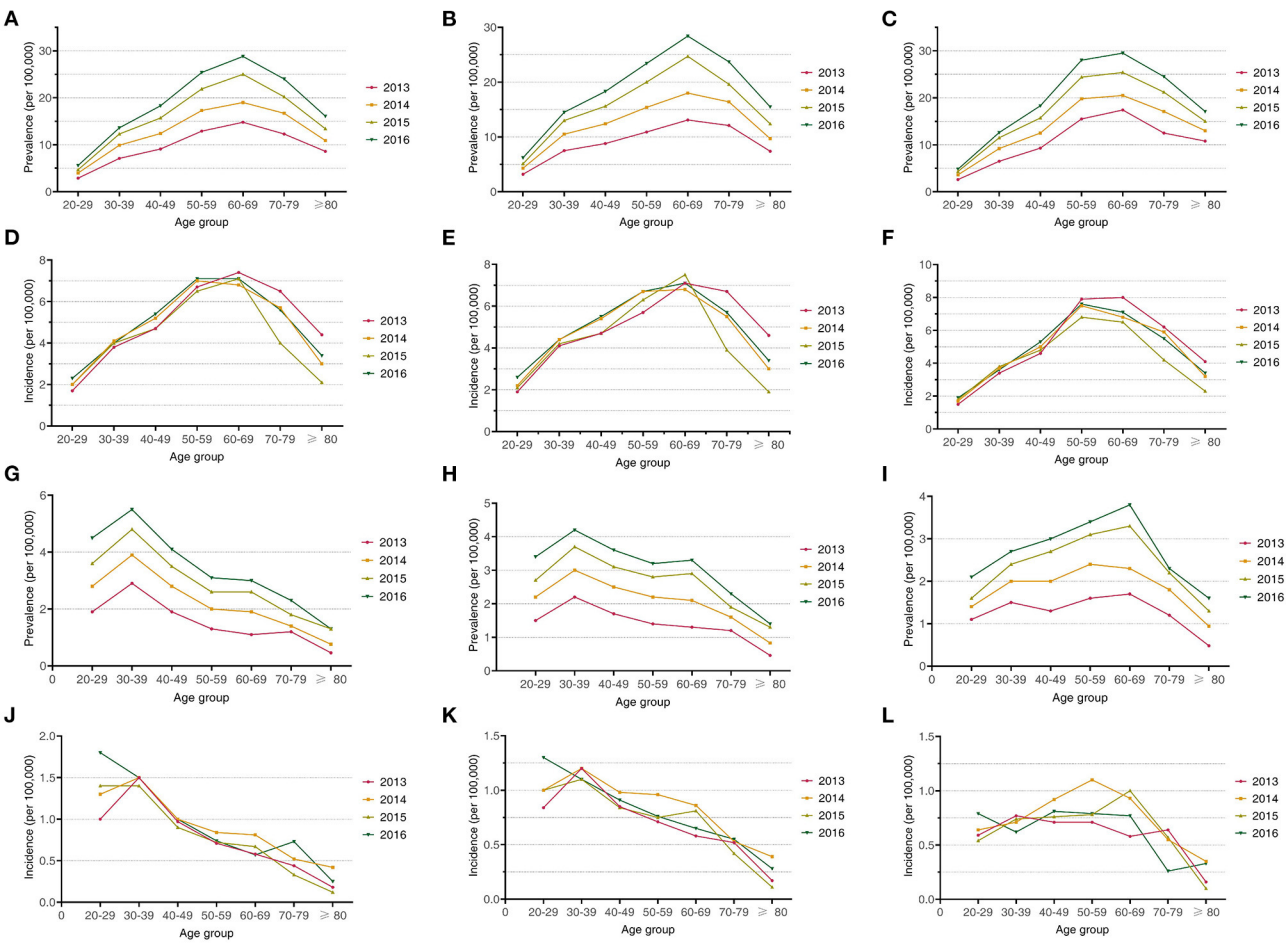
**(A)** The prevalence of UC patients from different age groups; **(B)** the prevalence of male UC patients from different age groups; **(C)** the prevalence of female UC patients from different age groups; **(D)** the incidence of UC patients from different age groups; **(E)** the incidence of male UC patients from different age groups; **(F)** the incidence of female UC patients from different age groups; **(G)** the prevalence of CD patients from different age groups; **(H)** the prevalence of male CD patients from different age groups; **(I)** the prevalence of female CD patients from different age groups; **(J)** the incidence of CD patients from different age groups; **(K)** the incidence of male CD patients from different age groups; and **(L)** the incidence of female CD patients from different age groups.

In UC, the prevalence increased among patients of different age groups (Figures 1A–C). The prevalence of UC peaked at the range of 60–69 years and declined gradually. As age increased, the age-specific incidence grew to a peak in the 50–69-year age group and then decreased gradually. Through the analysis by sex and age, we found that the peak of UC incidence among women shifted from the age group of 60–69 years to 50–59 years, while the peak among men remained at the age group of 60–69 years (Figures 1D–F).

In CD, the overall prevalence increased over time across all age groups in both men and women (Figures 1G–I). During the 4-year study period, the prevalence of CD peaked in the age range of 30–39 years and then declined gradually. A sex disparity in the age-specific prevalence was observed, with the prevalence of CD peaking at 30–39 years in men and from 60 to 69 years in women. The age pattern for incidence differed by sex and changed over time (Figures 1J–L). In male patients diagnosed between 2013 and 2015, the peak age range of diagnosis was 30–39 years, while in male patients diagnosed in 2016, the peak age range shifted downward to 20–29 years. The peak age range of CD in female patients shifted upward gradually from 30–39 years in 2013 to 50–59 years in 2014 and 60–69 years in 2015, and then back down to 40–49 years in 2016.

### Geographic Disparities of IBD in Prevalence and Incidence

The prevalence of UC by region was analyzed by stratifying geographic areas as eastern, central, and western China, and details of the prevalence cases are shown in Supplementary Table 1. Increasing rates of prevalence of UC were obvious in all three regions. The crude prevalence rate of UC in the eastern region was 1.64 times, 0.08 times for the western region, and 0.14 times for the central region compared with the total crude prevalence in 2016 (Table 9). As to the age-standardized prevalence, from 2013 to 2016, the eastern region had the highest prevalence of UC (*p* < 0.05), and then, the central and western regions, and eastern China also had the highest annual growth rate at 28.50% (95% CI: 14.20–44.60%, *p* < 0.05).

**Table 9:** Annual crude and age-standardized prevalence rate of ulcerative colitis among urban employees in three regions of China from 2013 to 2016 (per 100,000 persons).

	**Eastern region**		**Central region**		**Western region**	
	**Crude**	ASR^*^	**Crude**	**ASR**	**Crude**	**ASR**
2013	12.00 (11.70–12.30)	13.90 (13.58–14.22)	1.40 (1.20–1.60)	1.39 (1.20–1.58)	0.79 (0.69–0.92)	0.82 (0.70–0.94)
2014	17.40 (17.10–17.70)	19.69 (19.34–20.05)	1.80 (1.60–2.00)	1.76 (1.58–1.93)	1.20 (1.10–1.30)	1.21 (1.07–1.34)
2015	22.60 (22.30–23.00)	25.67 (25.27–26.07)	1.90 (1.70–2.10)	1.87 (1.69–2.04)	1.30 (1.20–1.50)	1.27 (1.14–1.40)
2016	27.20 (26.80–27.50)	30.65 (30.24–31.06)	2.40 (2.20–2.50)	2.32 (2.14–2.50)	1.30 (1.20–1.40)	1.26 (1.14–1.38)
APC^**^ (%)	28.50 (14.20–44.60)		16.90 (5.50–29.70)		11.20 (−15.40–46.30)	

* *Age-standardized rate*.

** *Annual percentage change*.

We also estimated the crude incidence of UC by region (a detailed number of cases were shown in Supplementary Table 1). The crude incidence per 100,000 appeared to increase in eastern China from 6.20 (95% CI: 6.00–6.40) to 7.60 (95% CI: 7.40–7.80); in central China from 0.90 (95% CI: 0.76–1.10) to 0.99 (95% CI: 0.88–1.10); and in western China from 0.59 (95% CI: 0.50–0.70) to 0.61 (95% CI: 0.45–0.70) (Table 10). The incidence rate of UC in the eastern region was 1.62 times, along with 0.21 times for the western region and 0.13 times for the central region, compared with the total incidence in 2016. The age-standardized incidence by region showed the same trend as did the region-specific crude prevalence, and eastern China had the significantly highest incidence (*p* < 0.05) and the highest annual average change rate of 4.50% (95% CI: −4.00 to 13.60%, *P* = 0.2) among the three regions.

**Table 10:** Annual crude and age-standardized incidence rate of ulcerative colitis among urban employees in three regions of China from 2013 to 2016 (per 100,000 persons).

	**Eastern region**		**Central region**		**Western region**	
	**Crude**	ASR^*^	**Crude**	**ASR**	**Crude**	**ASR**
2013	6.20 (6.00–6.40)	7.10 (6.88–7.33)	0.90 (0.76–1.10)	0.89 (0.74–1.04)	0.59 (0.50–0.70)	0.61 (0.51–0.72)
2014	6.90 (6.70–7.10)	7.56 (7.35–7.78)	0.97 (0.85–1.10)	0.94 (0.82–1.07)	0.65 (0.56–0.75)	0.66 (0.56–0.76)
2015	6.70 (6.60–6.90)	7.34 (7.13–7.55)	0.81 (0.71–0.94)	0.79 (0.68–0.91)	0.54 (0.46–0.63)	0.52 (0.44–0.60)
2016	7.60 (7.40–7.80)	8.25 (8.04–8.46)	0.99 (0.88–1.10)	0.97 (0.85–1.09)	0.61 (0.54–0.70)	0.61 (0.52–0.69)
APC^**^ (%)	4.50 (−4.00–13.60)		1.40 (−18.50–26.20)		−2.60 (−22.10–21.70)	

* *Age-standardized rate*.

** *Annual percentage change*.

Overall, CD prevalence rates increased over time across all three regions of China (Table 11). The prevalence of CD varied greatly among three regions, with the highest rate seen in the eastern region and the lowest rate seen in the western region. Similarly, age-standardized prevalence rates by region showed wide geographic disparities. In 2016, the age-standardized prevalence rate was highest in the eastern region (5.30/100,000, 95% CI: 5.14–5.46/100,000), followed by the central region (1.45/100,000, 95% CI: 1.31–1.60/100,000), and finally the western region (0.25/100,000, 95% CI: 0.18–0.31/100,000). However, the changing rate of age-standardized prevalence was highest in the western region, with an average of 40.70% per year (95% CI: −27.10 to 171.50%, *P* = 0.1).

**Table 11:** Annual crude and age-standardized prevalence rate of Crohn's disease among urban employees in three regions of China from 2013 to 2016 (per 100,000 persons).

	**Eastern region**		**Central region**		**Western region**	
	**Crude**	ASR^*^	**Crude**	**ASR**	**Crude**	**ASR**
2013	2.40 (2.30–2.50)	2.33 (2.21–2.45)	0.71 (0.59–0.86)	0.70 (0.56–0.83)	0.05 (0.03–0.09)	0.05 (0.02–0.08)
2014	3.50 (3.40–3.60)	3.43 (3.29–3.57)	1.10 (0.99–1.30)	1.12 (0.97–1.26)	0.16 (0.12–0.22)	0.17 (0.12–0.22)
2015	4.60 (4.40–4.70)	4.46 (4.31–4.62)	1.20 (1.10–1.40)	1.25 (1.11–1.40)	0.21 (0.16–0.27)	0.21 (0.15–0.26)
2016	5.40 (5.30–5.60)	5.30 (5.14–5.46)	1.50 (1.30–1.60)	1.45 (1.31–1.60)	0.22 (0.18–0.28)	0.25 (0.18–0.31)
APC^**^ (%)	29.30 (11.70–49.60)		21.90 (−3.30–53.50)		40.70 (−27.10–171.50)	

* *Age-standardized rate*.

** *Annual percentage change*.

As shown in Table 12, the crude incidence rates also varied substantially by region. Among the three regions in China, the incidence rates were highest in the eastern region and lowest in the western region. Crude incidence of CD in the western region appeared to increase over time during the study period, from 0.04/100,000 in 2013 to 0.10/100,000 in 2016. Also, as shown in Table 12, the eastern region had the highest age-standardized incidence, followed by the central region and western region from 2013 through 2016. The APC in the western region was 5.10% (95% CI: −7.20 to 19.10%, *P* = 0.8).

**Table 12:** Annual crude and age-standardized incidence rate of Crohn's disease among urban employees in three regions of China from 2013 to 2016 (per 100,000 persons).

	**Eastern Region**		**Central Region**		**Western Region**	
	**Crude**	ASR^*^	**Crude**	**ASR**	**Crude**	**ASR**
2013	1.20 (1.10–1.30)	1.17 (1.08–1.25)	0.43 (0.34–0.55)	0.43 (0.32–0.53)	0.04 (0.02–0.08)	0.05 (0.02–0.08)
2014	1.40 (1.30–1.50)	1.36 (1.27–1.44)	0.65 (0.55–0.77)	0.65 (0.54–0.76)	0.11 (0.08–0.16)	0.12 (0.07–0.17)
2015	1.30 (1.30–1.40)	1.27 (1.18–1.35)	0.37 (0.30–0.46)	0.38 (0.29–0.46)	0.10 (0.07–0.14)	0.09 (0.05–0.13)
2016	1.50 (1.40–1.50)	1.42 (1.33–1.50)	0.49 (0.41–0.58)	0.48 (0.40–0.57)	0.10 (0.07–0.14)	0.13 (0.08–0.18)
APC^**^ (%)	5.10 (−7.20–19.10)		−4.60 (−47.40–73.10)		22.60 (−41.80–158.30)	

* *Age-standardized rate*.

** *Annual percentage change*.

## Discussion

In this study, we reported the incidence and prevalence rates and trends of IBD from 2013 to 2016 using the NUEBMI database that provided comprehensive data from 23 provinces (municipalities and autonomous regions) of China, accounting for ~10% of the Chinese population. To the best of our knowledge, this study is the first and largest population-based study of IBD incidence and prevalence in China. In this study, we observed a significant increasing trend of IBD prevalence from 2013 to 2016 with a 21.0% APC for CD and 24.2% for UC, while the incidence rate did not significantly change during the 4-year study period either for CD or UC. In China, the prevalence and incidence of UC were higher than CD with the ratio of UC to CD at 5.09 for prevalence and 5.11 for incidence, respectively, in 2016. The ratio of UC to CD had a decreasing trend within 4 years. Among the three regions of China, the eastern region had the highest prevalence and incidence rate for IBD, while the western region had the lowest prevalence and incidence. It exhibited the gradient between east and west.

The majority of IBD surveys have been conducted in European countries, and data are lacking on the population-based incidence and prevalence of IBD in developing countries. This study provided systematic data to describe the overall prevalence and incidence of IBD in China and the population stratified by gender and age groups. The crude incidence from this study was higher than that reported from Daqing (1.86 in total, 1.82 for men, and 1.90 for women, per 100,000 for UC; 0.15/100,000 in total for CD in 2011–2012) (4), Wuhan (1.59 in total, 1.28 for men, 1.63 for women for UC; 0.56 in total, 0.49 and 0.52 for men and women, respectively, for CD in 2011–2012) (14), and Hong Kong (1.51 for UC and 1.46 in for CD in 2014) (8). The prevalence of IBD determined by this study was lower than that of Hong Kong (30 for men, 20 for women per 100,000 for UC and 27 for men, and 12 for women per 100,000 for CD in 2014) (8). The epidemiologic differences between these reports are attributed to the different study populations and regions enrolled. Recently, a Bayesian meta-regression model-based IBD burden research in China also revealed increasing trends in prevalence (APC: 2.9%) and incidence (APC: 1.1%) from 1990 to 2017, and this statistical model-based estimation needed validation through a nationwide study (15). The results in this study testified to the feasibility to estimate IBD prevalence/incidence using NUEBMI, which may be used to validate the model-based estimation.

The incidence and prevalence of IBD are highly variable across the world. The highest estimated incidence of UC was 57.9 per 100,000 (Faroe Islands, nationwide), and the highest estimated prevalence of UC was 505 per 100,000 (Southeast, Norway), both in Northern Europe. Nova Scotia in Canada has the highest estimated incidence of CD (29.3/100,000) (16), and Hesse in Germany has the highest estimated prevalence of CD (322/100,000) (17). The highest incidence of UC in East Asia was 4.6 per 100,000 [Seoul, South Korea (10)], and the highest prevalence of UC was 57.3 per 100,000 [Japan, Nationwide (18)]. The highest incidence of CD was reported to be 3.2/100,000 in South Korea in an observational study conducted between 2006 and 2012 (10), and the highest prevalence of CD was reported to be 18.6/100,000 in Japan (18).

In addition, we found that the overall incidence appeared to fluctuate over the years included in the analysis. However, a significant finding of this analysis was the rising trend of IBD prevalence. The crude prevalence of IBD more than doubled over 4 years, and the age-standardized prevalence increased at an annual rate of 21.1% for CD and 22.4% for UC. Combined with the development trend of IBD in this study, IBD is going into the rank of a common disease in China. This analysis also showed that the incidence of UC in China is numerically approaching the highest incidence of IBD in East Asia. The difference in IBD incidence between China and Europe may be related to different risk factors such as speed of industrialization, urbanization, and Western lifestyle (3). The rising prevalence of IBD with a stable incidence rate may be explained by factors that improve survival and prolong the duration of the disease. IBD is a chronic disorder that is considered incurable (19, 20). Recent advances in the diagnosis and clinical management of IBD may change the course of disease progression. Specifically, with the publication and promotion of a standardized diagnosis and treatment for CD, the pre-diagnostic period has dramatically decreased from 39.4 months in 2010 to 3.1 months in 2015 (21), and the mortality of hospitalized patients with CD decreased from 3.8% in 1996–2000 to 1.61% in 2010–2015 (22, 23). Furthermore, a registry study from several medical centers in China also revealed that risk factors of typical clinical outcomes in CD patients, for example, older age, longer disease course, structuring behavior, penetrating behavior, and perianal disease, were associated with surgery (17), which highlighted the necessity to look for risk factors of IBD in China from a larger scale of study in the future.

In this study, it was shown that eastern China had the most patients compared with central or western China. In addition, the IBD burden had the fastest increasing rate in eastern China, as reflected by both prevalence and incidence. This study enhances our understanding of the geographic heterogeneity of IBD in China and could eventually assist in designing preventive strategies to control this disease. Global studies have confirmed that there are obvious geographic variations in IBD incidence (24, 25). The variation in the incidence of IBD among regions may partly be explained by varying risk factors related to, e.g., the socio-economy, health resources, and traditional diets. A small-scale research about GDP and CD prevalence in 23 provinces in China found a positive correlation between regional prevalence and economic development level, so the three regions in this study were also grouped based on the levels of economic development (26). Besides, a prospective population-based study in 13 countries/regions in Asia-Pacific also witnessed a west-to-east gradient for CD incidence in China (higher incidence in the east) and revealed that GDP was significantly associated with IBD incidence (IRR of IBD: 2.37, 95% CI: 1.09–5.16) (3). The original data from the State Statistical Bureau (27) indicate that the eastern region represents an important economically developed region with both traditional and emerging technological industries. The central region focuses on energy development. The economy of western China rests on agriculture, forestry, and transportation, with lower gross domestic product. The national statistics showed that the eastern region had the highest gross domestic product and the most rapid economic development (28), leading to faster urbanization, where people bear a higher burden of UC. Thus, in eastern China, especially in developed cities, more healthcare assignments would be urgently needed to improve the diagnostic level of IBD. Further study to evaluate the different influences of medical capacity for IBD diagnosis and treatment in various regions should be carried out for a better understanding of the disease burden among regions.

The prevalence and incidence of IBD were higher in male patients when compared with female patients across all age groups. A study in Canada reported a higher incidence in female patients than in male patients (25), but CD registration research in Mainland China (21) and Hong Kong (8) has reported that CD is more prevalent in male patients than in female patients in China. The gender differences may be associated with smoking, endocrine-related factors, and genetic factors, among others (29). In China, the prevalence of smoking in male patients (50%) was significantly higher than that in female patients (11%) according to the 2015 China Adult Tobacco Survey Report (30, 31), and some meta-analyses have shown that individuals who smoked had a 1.76-fold risk to develop CD than non-smokers (32, 33).

This study has several limitations. A common problem in a medical insurance database is the accuracy of diagnosis. To control bias caused by diagnostic errors, we only included patients with the diagnostic code K50 or K51 claiming at least three times for outpatients and inpatients. In addition, according to the principle of insurance reimbursement, all patients must have a defined diagnosis before reimbursement can be given. Also, our study used only the NUEBMI database, which only represented the urban working and retired employees whose distribution of IBD-related risk factors may differ from those of rural population and urban residents who did not work. Admittedly, Beijing and Shanghai, two highly industrialized cities, were excluded from our study due to a lack of data, which may result in the underestimation of nationwide UC prevalence or incidence. Last but not least, due to patient confidentiality concerns, we had limited assessment of raw recordings from the NUEBMI system, so we could not analyze risk factors on IBD in China for the time being.

We observed a rising trend of IBD prevalence in most urban regions of China and found a marked east-to-west gradient in CD incidence and prevalence. Efforts to understand regional-specific environmental risk factors that drive the occurrence of IBD are needed. Our findings highlight the importance of developing and targeting prevention strategies by geographic regions, particularly in young men who are most “at risk” of developing CD. In addition, the decreasing trend of the ratio of UC to CD in China is similar to the worldwide trend, which suggests we should pay great attention to the increasing possibility of CD, although the incidence of CD is not high now. The increasing prevalence of IBD highlights the need to improve preparedness with healthcare services and medical insurance. Moreover, a national etiologic factor survey should be conducted to determine the risk factors that are strongly associated with IBD in China.

## Data Availability Statement

The original contributions presented in the study are included in the article/Supplementary Material, further inquiries can be directed to the corresponding author/s.

## Author Contributions

HY, LW, and JQ: study design, data collection, and manuscript preparation. RZ: statistics analysis. GR, MG, and XB: assistance in data collection and critical comment. All authors have read and approved the final manuscript.

## Funding

This study was supported by the National Natural Science Foundation of China [grant number 81570505, 81970495], the Natural Science Foundation of Beijing Municipality [grant number 7202161], the CAMS Innovation Fund For Medical Science [grant number 2016-I2M-3-001, 2019-I2M-2-007], and the National Health and Family Planning Commission of the People's Republic of China [grant number 201502005].

## Conflict of Interest

The authors declare that the research was conducted in the absence of any commercial or financial relationships that could be construed as a potential conflict of interest.

## Publisher's Note

All claims expressed in this article are solely those of the authors and do not necessarily represent those of their affiliated organizations, or those of the publisher, the editors and the reviewers. Any product that may be evaluated in this article, or claim that may be made by its manufacturer, is not guaranteed or endorsed by the publisher.

## Supplementary Material

Supplementary Figure 1 **(A–D)** Composition of 2013–2016 NUEBMI and Census 2015 population.

Supplementary Figure 2 **(A)** The standardized prevalence of UC patients in different calendar years; **(B)** the standardized prevalence of CD patients in different calendar years; **(C)** the standardized incidence of UC patients in different calendar years; **(D)** the standardized incidence of CD patients in different calendar years.
