# Supplementary material for: Trend and Geographic Variation in Incidence and Prevalence of Inflammatory Bowel Disease in Regions Across China: A Nationwide Employee Study Between 2013 and 2016
Source: Front Med (Lausanne). 2022 Jul 25;9:900251. doi: 10.3389/fmed.2022.900251 (PMC9357923; doi:10.3389/fmed.2022.900251)
Supplement: Supplementary file 1 [file Table_1.docx]

|  | Eastern region | | | Central region | | | Western region | | |
| --- | --- | --- | --- | --- | --- | --- | --- | --- | --- |
|  | insured cases | incidence cases | prevalence cases | insured cases | incidence cases | prevalence cases | insured cases | incidence cases | prevalence cases |
| CD |  |  |  |  |  |  |  |  |  |
| 2013 | 69120720 | 844 | 1659 | 15020910 | 65 | 107 | 22644906 | 10 | 12 |
| 2014 | 77445093 | 1073 | 2716 | 21851139 | 142 | 244 | 27070606 | 31 | 44 |
| 2015 | 79965833 | 1072 | 3651 | 24334770 | 91 | 304 | 29180602 | 28 | 61 |
| 2016 | 87973859 | 1290 | 4794 | 27729386 | 135 | 410 | 34914480 | 36 | 77 |
| UC |  |  |  |  |  |  |  |  |  |
| 2013 | 69120720 | 4282 | 8293 | 15020910 | 135 | 210 | 22644906 | 134 | 180 |
| 2014 | 77445093 | 5351 | 13460 | 21851139 | 213 | 392 | 27070606 | 176 | 321 |
| 2015 | 79965833 | 5384 | 18108 | 24334770 | 198 | 462 | 29180602 | 158 | 386 |
| 2016 | 87973859 | 6659 | 23885 | 27729386 | 274 | 652 | 34914480 | 214 | 452 |

Supplement Table 1. Number of insured cases, incidence cases and prevalence cases of Crohn’s disease (CD) and Ulcerative colitis (UC) among urban employees by three regions of China from 2013 to 2016
