# Supplementary figures and images for: Trend and Geographic Variation in Incidence and Prevalence of Inflammatory Bowel Disease in Regions Across China: A Nationwide Employee Study Between 2013 and 2016
Source: Front Med (Lausanne). 2022 Jul 25;9:900251. doi: 10.3389/fmed.2022.900251 (PMC9357923; doi:10.3389/fmed.2022.900251)

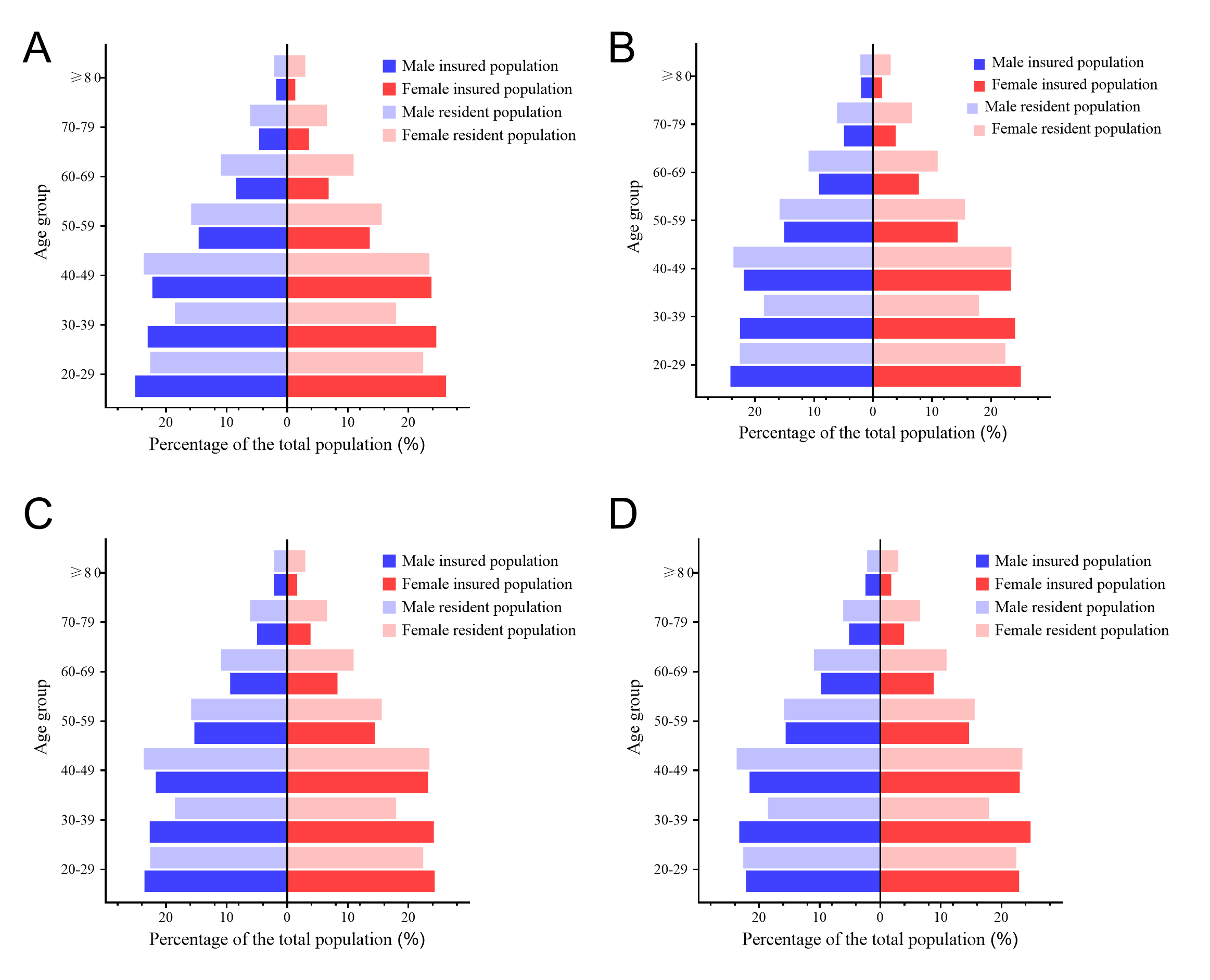

Supplement: Supplementary Figure 1 — (A–D) Composition of 2013–2016 NUEBMI and Census 2015 population. [file Image_1.TIF]

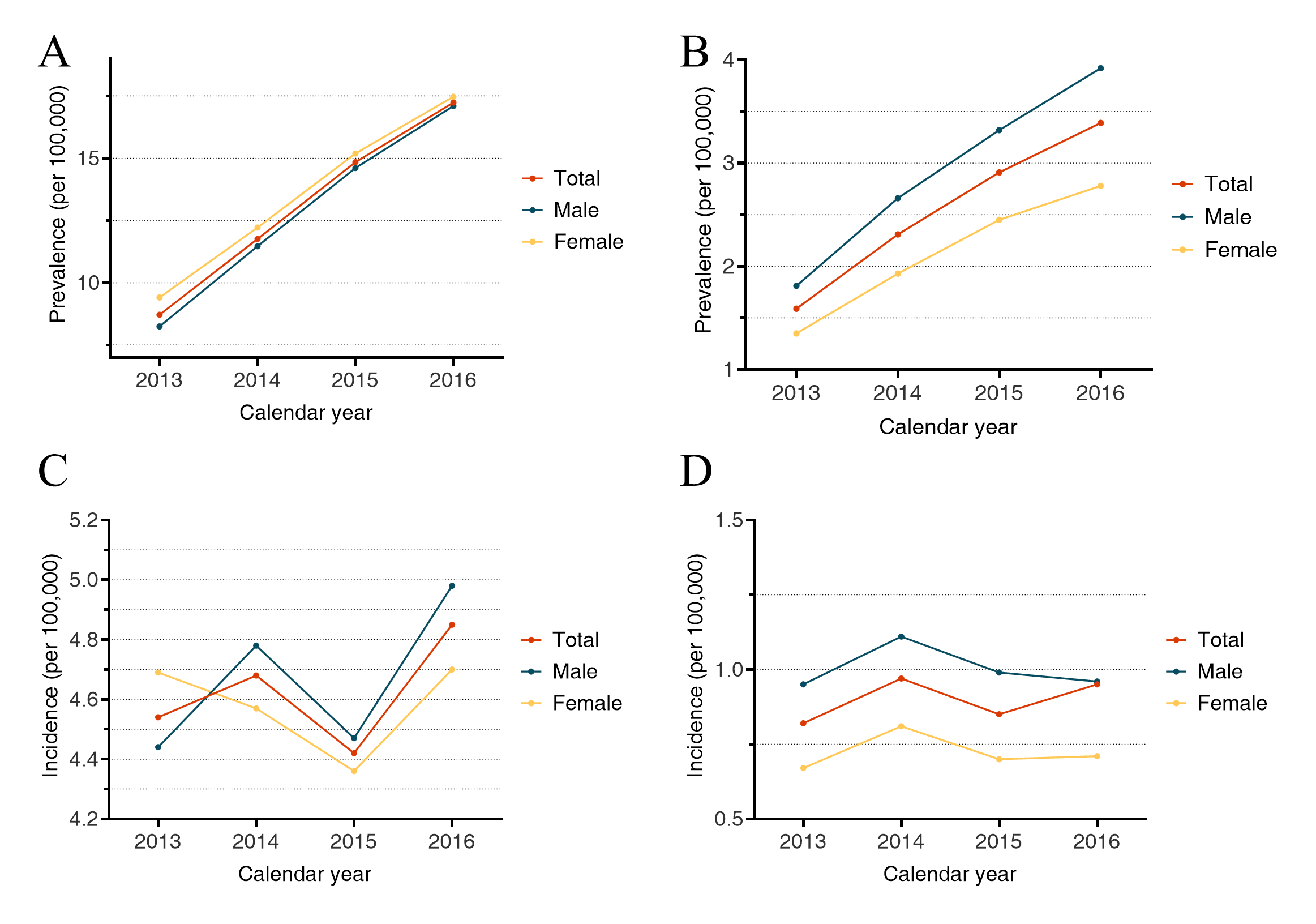

Supplement: Supplementary Figure 2 — (A) The standardized prevalence of UC patients in different calendar years; (B) the standardized prevalence of CD patients in different calendar years; (C) the standardized incidence of UC patients in different calendar years; (D) the standardized incidence of CD patients in different calendar years. [file Image_2.TIF]
